# Hexaminolevulinate Blue-Light Cystoscopy in a Patient with Metastatic Melanoma of the Bladder

**DOI:** 10.1089/cren.2016.0031

**Published:** 2016-04-01

**Authors:** Anant Shukla, Jonathan T. Wingate, Karen C. Baker, Timothy C. Brand

**Affiliations:** ^1^ Department of Urology, Uniformed Services University of the Health Sciences, F. Edward Hebert School of Medicine, Bethesda, Maryland.; ^2^ Department of Urology, Madigan Army Medical Center, Tacoma, Washington.

## Abstract

***Background:*** Although bladder cancer is one of the most frequently diagnosed tumors worldwide, metastatic melanoma of the bladder is a rare occurrence with only 29 cases reported in the literature.

***Case Presentation:*** We present the case of a 60-year-old male with a medical history significant for metastatic melanoma, who was referred to the urology department for gross hematuria. Transurethral resection of bladder tumor (TURBT) was performed with the assistance of hexaminolevulinate acid (HAL) with blue-light cystoscopy (BLC). Subsequent histopathologic analysis of the specimen confirmed a diagnosis of metastatic melanoma of the bladder. To our knowledge, this is the first reported case of metastatic bladder melanoma diagnosed with the assistance of HAL-BLC in a patient undergoing a TURBT.

***Conclusion:*** Although HAL-BLC is only indicated for use in the cystoscopic detection of papillary nonmuscle invasive bladder cancer, it may aid in the detection of nonconventional bladder pathologies, such as melanoma.

## Case

**FIG. 1.**
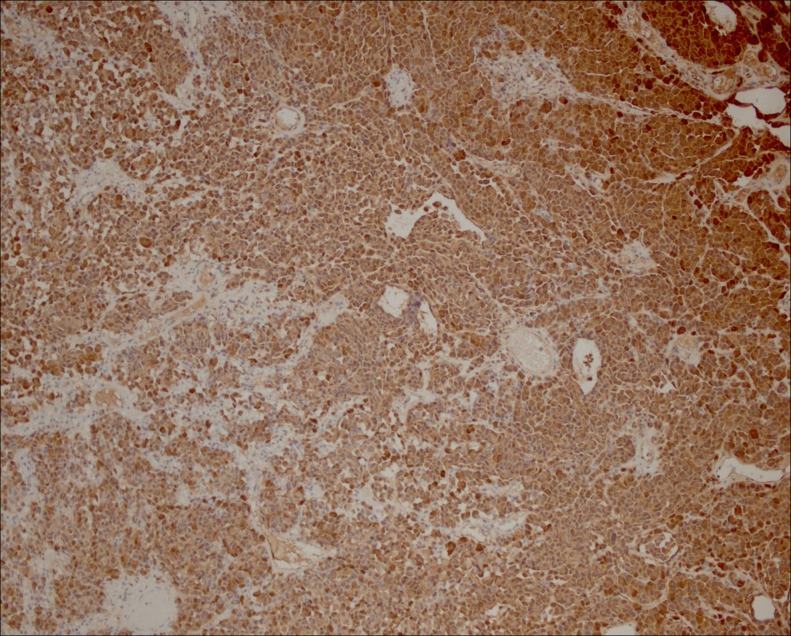
Immunohistochemistry shows strong positivity for S100 on lesional cells.

**FIG. 2.**
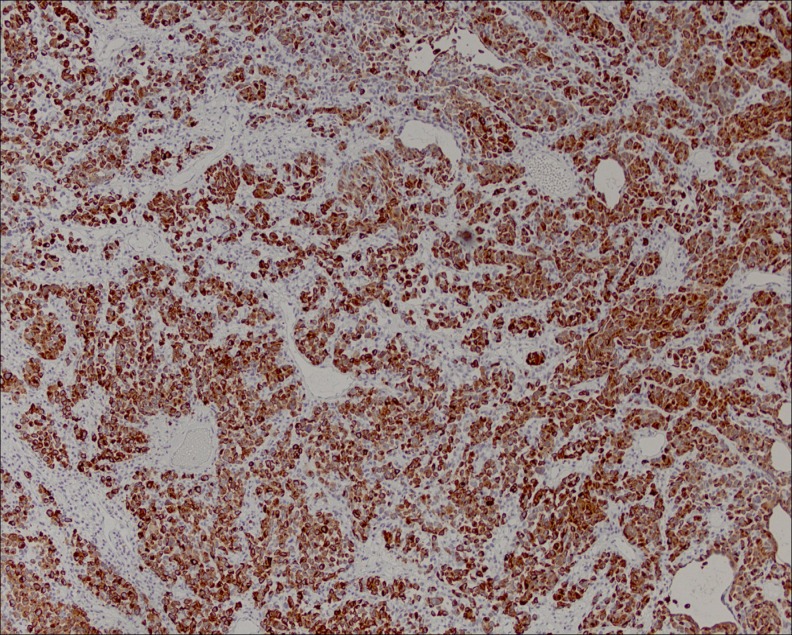
Strong cytoplasmic positivity with melanocytic markers (MART-1).

A 60-year-old Caucasian male presented to our urology clinic with a chief complaint of gross hematuria. The patient's medical history was significant for a diagnosis of malignant melanoma of the left distal thigh in 2003, which was treated with wide local excision, sentinel node biopsy, and left groin dissection. A positron emission tomography scan in 2007 showed increased lymph node activity in the neck, along with retroperitoneal and left inguinal lymphadenopathy. In 2008, he underwent isolated limb perfusion with melphalan and actinomycin D, and demonstrated a partial response. After being lost to follow-up, he returned to our hospital system in 2014 with metastatic melanoma, with a Clark III pectoral lesion along with a new brain lesion. He was started on pembrolizumab treatment to which he showed a partial response. In 2015 he presented to the urology clinic with a one-and-a-half-month history of painless gross hematuria. Cystoscopy showed a 2 cm papillary tumor in the left lateral wall of the bladder and it was fluorescent under hexaminolevulinate acid with blue-light cystoscopy (HAL-BLC). After a successful transurethral resection of bladder tumor (TURBT) of the lesion, he received 40 mg of intravesical mitomycin-C postoperatively. Pathologic review of the specimen along with histochemical analysis using melanoma-specific stains, S-100 (Fig. 1) and melanocytic antigen recognized by cytotoxic T lymphocytes (MART-1) (Fig. 2), supported a diagnosis of metastatic melanoma of the bladder. The patient is still alive and continues to seek care at a tertiary medical facility.

## Discussion

Metastatic melanoma of the bladder is a rare occurrence in clinical practice with only 29 other cases of this diagnosis reported in the literature. An autopsy series of metastatic melanoma patients demonstrated that 18% of the subjects had metastatic melanoma of the bladder, indicating that many patients with this condition remain undiagnosed.^1^ Symptomatic metastatic melanoma of the bladder carries a very poor prognosis with our review demonstrating that most patients died within a year of being diagnosed. Patients will typically present with gross hematuria and a history of malignant melanoma that may predate the onset of urinary symptoms by months to years.^2^

HAL-BLC has been shown in randomized controlled trials to improve detection and reduce recurrence of nonmuscle invasive bladder cancer (NMIBC) when compared with white-light cystoscopy (WLC).^3^ While the Food and Drug Administration has approved HAL-BLC for NMIBC tumor detection, our case demonstrates that there may be a role for this therapeutic adjunct in rarer nonurothelial cell carcinoma (UCC) tumors such as melanomas. Although it is difficult to predict which other types of rare non-UCC tumors may also be detected with HAL-BLC, with more published reports of non-UCC diagnoses made with the assistance of HAL-BLC, there can be a better appreciation of HAL-BLC's full application.

**FIG. 3.**
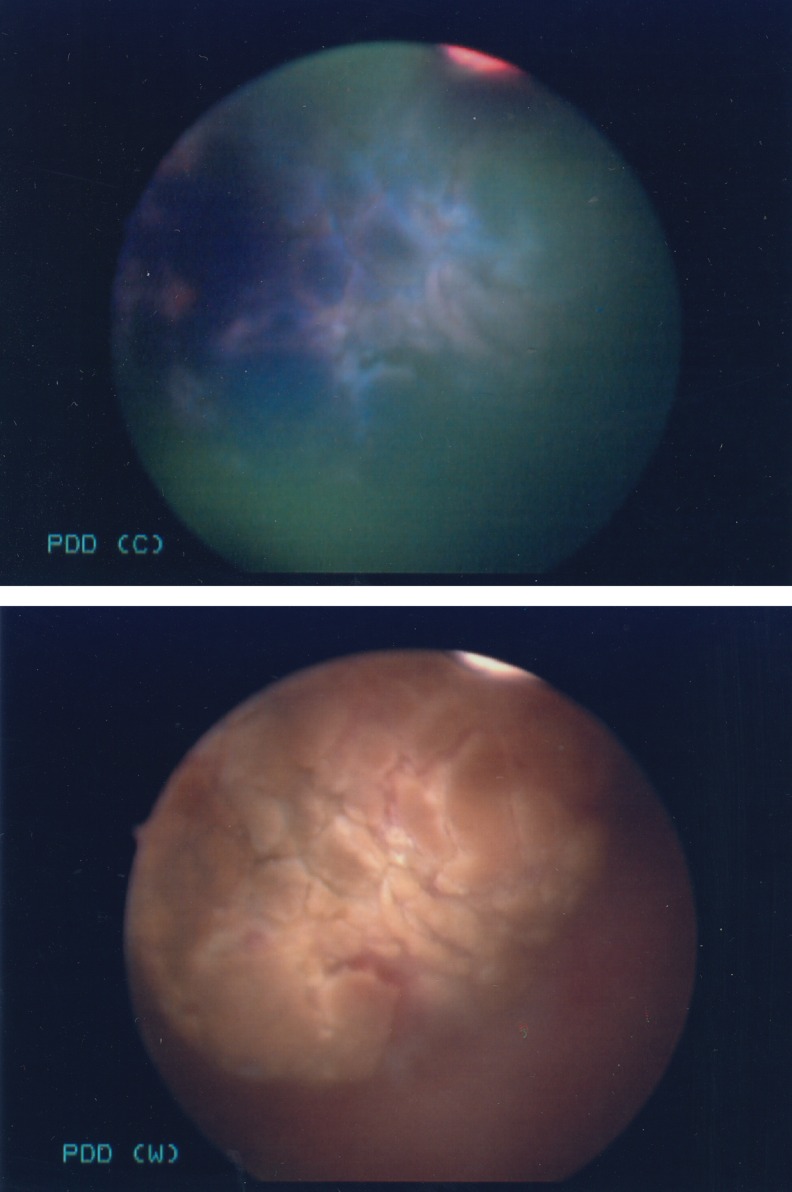
Appearance of bladder tumor in HAL-BLC versus white-light cystoscopy.

HAL induces the accumulation of porporynin IX within rapidly proliferating cells, which allows it to be distinguished from nonmalignant tissues under BLC. With better observability, HAL has allowed surgeons to perform more complete TURBTs and significantly reduce NMIBC recurrences when compared with WLC alone.^3^ Figure 3 shows the 2 cm bladder tumor from this case under both BLC and WLC, with the tumor fluorescing vividly under blue light, facilitating a complete resection. Although HAL-BLC has not demonstrated to change progression of UCC disease, it has been shown in small published studies to change recurrence and progression risk categories in patients when used as an adjunct to WLC. The change in risk categories alters the subsequent postoperative management in determining whether a patient will receive Bacillus Calmette-Guerin for high-risk disease, intravesical mitomycin-C for intermediate risk disease, or no postoperative treatment for low-risk disease.^4^

To our knowledge, this is the first published case of metastatic bladder melanoma diagnosed with the assistance of HAL-BLC.

## Conclusions

Although HAL-BLC is only indicated for use in the cystoscopic detection of papillary NMIBC, it may aid in the detection of nonconventional bladder pathologies, such as melanoma. It has been shown to be well tolerated and effective in improving detection and reducing the recurrence rates of NMIBC. Although metastatic melanoma of the bladder carries a very poor prognosis, improved detection may facilitate earlier intervention and a more complete resection.

## Abbreviations Used

BLC: blue-light cystoscopy
HAL: hexaminolevulinate acid
MART-1: melanocytic antigen recognized by cytotoxic T lymphocytes
NMIBC: nonmuscle invasive bladder cancer
TURBT: transurethral resection of bladder tumor
UCC: urothelial cell carcinoma
WLC: white-light cystoscopy
